# Establishment of In Vitro and In Vivo Anticolorectal Cancer Efficacy of Lithocholic Acid-Based Imidazolium Salts

**DOI:** 10.3390/ijms23137019

**Published:** 2022-06-24

**Authors:** Diana Sawicka, Agnieszka Hryniewicka, Sylwia Gohal, Anna Sadowska, Anna Pryczynicz, Katarzyna Guzińska-Ustymowicz, Emilia Sokołowska, Jacek W. Morzycki, Halina Car

**Affiliations:** 1 Department of Experimental Pharmacology, Medical University of Bialystok, Szpitalna Street 37, 15-295 Bialystok, Poland; zfarmdosw@umb.edu.pl (S.G.); anna.sadowska@umb.edu.pl (A.S.); emiliasokolowska.umwb@gmail.com (E.S.); halina.car@umb.edu.pl (H.C.); 2 Department of Organic Chemistry, Medical University of Bialystok, Mickiewicza Street 2A, 15-222 Bialystok, Poland; agnieszka.hryniewicka@umb.edu.pl; 3 Department of General Pathomorphology, Medical University of Bialystok, Waszyngtona Street 13, 15-269 Bialystok, Poland; anna.pryczynicz@umb.edu.pl (A.P.); katarzyna.guzinska-ustymowicz@umb.edu.pl (K.G.-U.); 4 Department of Natural Product Chemistry, University of Bialystok, Ciołkowskiego Street 1K, 15-245 Bialystok, Poland; morzycki@uwb.edu.pl

**Keywords:** imidazolium salts, lithocholic acid, colorectal cancer

## Abstract

Imidazolium salts (IMSs) are the subject of many studies showing their anticancer activities. In this research, a series of novel imidazolium salts substituted with lithocholic acid (LCA) and alkyl chains of various lengths (**S1**–**S10**) were evaluated against colon cancer cells. A significant reduction in the viability and metabolic activity was obtained in vitro for DLD-1 and HT-29 cell lines when treated with tested salts. The results showed that the activities of tested agents are directly related to the alkyl chain length, where **S6**–**S8** compounds were the most cytotoxic against the DLD-1 line and **S4**–**S10** against HT-29. The research performed on the xenograft model of mice demonstrated a lower tendency of tumor growth in the group receiving compound **S6**, compared with the group receiving 5-fluorouracil (5-FU). Obtained results indicate the activity of **S6** in the induction of apoptosis and necrosis in induced colorectal cancer. LCA-based imidazolium salts may be candidates for chemotherapeutic agents against colorectal cancer.

## 1. Introduction

Colorectal cancer (CRC) is one of the most common malignant tumors in the world since 1950. About one million people are diagnosed with CRC each year, resulting in deaths in about half of the cases [1]. It is the cause of 8% of cancer deaths worldwide. The reasons for the increased morbidity are an aging population and the prevalence of bad eating habits, smoking, low physical activity, and obesity [2,3]. New treatments for primary and metastatic colorectal cancer have been developed so far that include laparoscopic surgery for primary disease, resection of metastatic disease affecting, radiotherapy for rectal cancer, and some forms of metastatic disease, as well as neoadjuvant and palliative chemotherapy [4,5]. 

Recently, the treatment of patients with colorectal cancer has developed owing to a significant improvement in adjuvant therapy. A typical chemotherapy backbone for first-line treatment of colorectal cancer is a combination of 5-fluorouracil (5-FU), leucovorin, and either oxaliplatin or irinotecan. It should be noted that strategies that aimed to improve by intensifying the chemoradiotherapy regimen (by combining 5-fluorouracil and oxaliplatin) have increased toxicity and have not exhibited clear survival benefits. Moreover, the available anticancer drugs cause numerous side effects and complications [6,7]. There is a need to further search for compounds with actions that would both inhibit the growth of cancer as well as prevent further relapses. 

In the present research, new lithocholic acid (LCA)-based imidazolium salts were tested. Imidazolium salts (IMSs) are the derivatives of imidazole that consist of discrete cation and anion pairs. The imidazole ring commonly occurs in nature and plays a crucial role in many structures of the human body via its ability to bond to metal ions as a ligand and also to form hydrogen bonds with proteins [8,9]. One of the most important properties of IMSs is the flexibility in the design of the physical, chemical, and biological property sets via the independent modification of the structure of cation and anion; therefore, they exhibit fungicidal and antibacterial activities [10,11]. Hydrophobic fragments of IMSs destroy the cell membrane, leading to the leakage of intracellular substances and, eventually, cell death. Recently, major consideration has focused on IMSs declared significant antitumor properties against human cancer cell lines through the induction of G1 phase cell cycle arrest and apoptosis [12,13]. Imidazolium salts are also used in bioengineering as drug carriers and biosensors. The biological properties of IMSs depend on their ionic structure as well as on various types of substituents attached to the nitrogen atoms of the imidazole ring [14,15]. The main objective of medicinal chemistry is to improve the biological properties of compounds, among other things, by combining bioactive molecules. It has been demonstrated that the connection of IMSs with other bioactive molecules improved the biological activities, including anticancer activities, of the resulting compounds, compared with those of the initial compounds [16,17,18]. 

LCA is an interesting pharmacological compound due to its antibacterial, antifungal activities, and inhibition of α-2,3-sialyltransferase [19,20,21,22,23]. The pharmacological interest in lithocholic acid is associated with the effective induction of vitamin D receptor (VDR) expression [24] and the demonstration of anticancer activity [25,26]. Epidemiological studies have suggested a protective role of vitamin D in the development of colorectal cancer [27]. Moreover, in vivo studies have shown that LCA effectively reduced the levels of proinflammatory cytokines such as TNF-α, IL-6, and IL-1 in the colonic mucosa. In addition, the administration of lithocholic acid to mice improved the protective functions of the colonic epithelium [28]. 

Our previous study revealed that steroid-derived imidazolium salts substituted with an alkyl chain of various lengths showed high activities against bacterial strains—namely, *Staphylococcus aureus*, *Bacillus cereus*, and *Escherichia coli* as well as pathogenic fungi of the genus *Candida albicans*, *Aspergillus niger*, *Aspergillus fumigatus*, *Trichophyton mentagrophytes*, and *Cryptococcus neoformans*—and phytopathogenic fungi, such as *Microsporum canis*, *Alternaria alternata*, *Botrytis cinerea*, *Cercospora beticola*, and *Fusarium culmorum* [29,30,31]. Moreover, the cytotoxicity assay showed that steroid-derived imidazolium salts only slightly inhibited the growth of healthy fibroblast (CRL-1475) and monocytic (THP-1) cells, which suggests their good compatibility with the host cells [31].

Due to the excellent activity of these new salts against bacteria and pathogenic fungi, as well as their lack of toxicity, we decided to subject them to anticancer tests. Therefore, LCA-based IMSs were examined for activity against colon cancer. The present paper, shows, for the first time, the anticancer potential of steroidal imidazolium salts depending on their chemical structure. 

## 2. Results and Discussion

### 2.1. Imidazolium Salts Decrease Colon Cancer Viability and Metabolic Activity 

Several reports have shown that imidazolium salts possess promising anticancer activities [13,16,32,33,34,35,36,37,38,39,40,41]. We evaluated the anticancer activities of lithocholic acid-based imidazolium salts (**S1**–**S10**) against three different colorectal cancer cell lines (DLD-1, HT-29, and Caco-2). DLD-1 and HT-29 are epithelial, colorectal adenocarcinoma cell lines characterized by positive expression of oncogenes such as myc, myb, ras, fos, sis, and p53 [42]. The results showed that there were differences in the cytotoxic effect depending on the structure of tested salts as well as applied concentrations (Table 1). The strongest reduction in DLD-1 cell viability was obtained for **S6** and **S8**. In the case of the HT-29 line, the cytotoxic effect of salts **S3**–**S10** was stronger than that of the DLD-1 line. The lowest IC50 value for HT-29 we obtained for compounds **S4** and **S8**–**S10**. In summary, the DLD-1 and HT-29 cell lines are sensitive to the tested imidazolium salts, depending on their chemical structure. 

The Biopharmaceutical Classification System has passed Caco-2 for standard methods for determining the bioavailability of drugs [43]. For this CRC line, we obtained the lowest reduction after the application of imidazolium salts (**S1**–**S10**), where the IC50 values were over threefold higher than those obtained for the DLD-1 and HT-29 lines. The strongest cytotoxic effect for Caco-2 cells was observed for **S7**. Interestingly, no correlation was found between the structure of the tested salts and their cytotoxic effect. Artursson et al. [44] showed that the transport of the particles through the Caco-2 layer is 20 times smaller than through the large intestine and about 100 times smaller than the human small intestine. Caco-2 cells have lesser gene expression of transport proteins [45]. This may explain the weaker influence of the tested imidazolium salts on the metabolic activity of these cells, compared with HT-29 and DLD-1 colon cancer cell lines.

Moreover, in previous studies, the cytotoxicity of tested imidazolium salts on normal fibroblasts cells CRL-1475 was assessed [31]. Besides the salt compounds **S3** and **S8**, inhibition on tested cell growth was lower than that of DLD-1 and HT-29 cells, which suggests good compatibility of the tested agent. The highest cytotoxic effect on fibroblasts viability was observed for compounds **S3** and **S8** (Table 1). 

**Figure 1:**
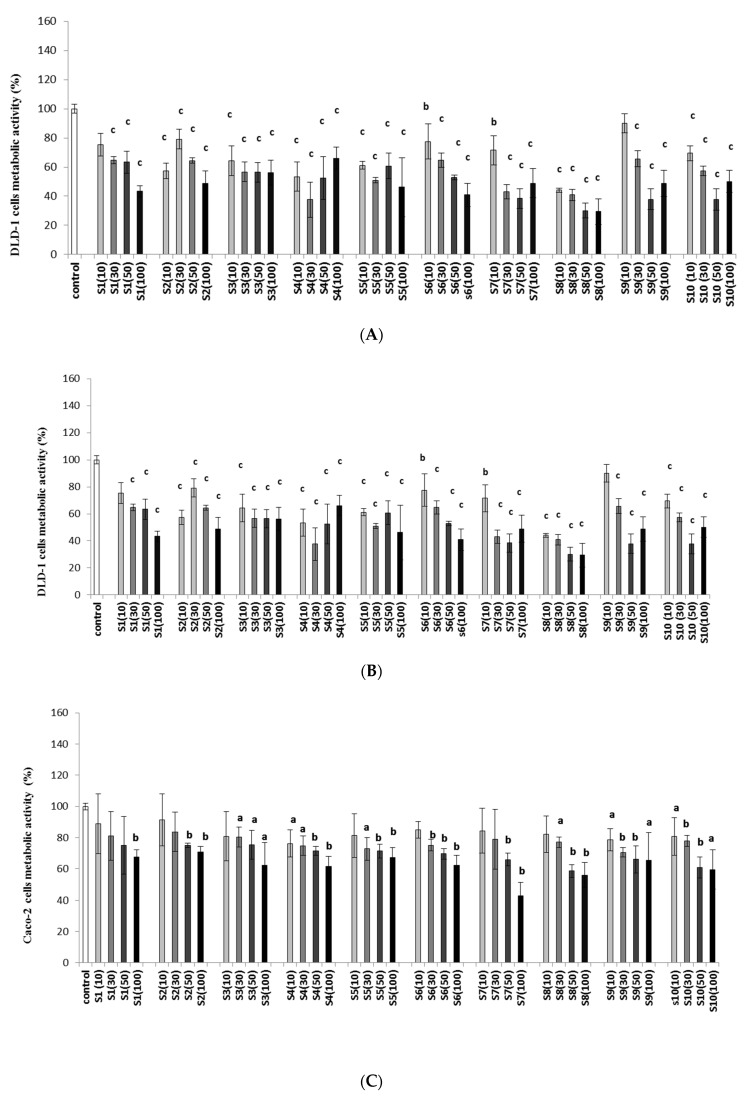
Effects of imidazolium salts (**S1**–**S10**) on the metabolic activity of DLD-1 (**A**), HT-29 (**B**), and Caco-2 (**C**) cells after 24 h of incubation. Concentrations (µg/mL) are given in parentheses. The results are presented as the mean values ±SD. **a** *p* < 0.05, **b** *p* < 0.01, and **c** *p* < 0.001, compared with the control.

To evaluate the mode of action of imidazolium salts, the metabolic activity was determined in the tested cell lines. The impact of tested imidazolium salts on the metabolic activity of cells showed that the tested agents decreased cell metabolism in all colon cancer cells (Figure 1A–C). A significant decrease in the metabolic activity of the DLD-1 line was noted after the application of all tested agents (Figure 1A). In the case of the HT-29 cell line (Figure 1B), a significant decrease in metabolic activity (*p* < 0.001) was observed only at the highest concentration (100 µg/mL) after exposure to **S1**. For compound **S2**, we did not observe significant depletion of metabolic activity in the HT-29 cell line. Imidazolium salts **S3–S10** at all concentrations, except for **S5** (10 µg/mL), significantly inhibited the metabolic activity of the HT-29 cell line. Importantly, the highest reduction in metabolic activity was observed in cells after administration of **S3** and **S6** at a concentration of 100 µg/mL.

The studies indicated that Caco-2 was the least sensitive colon cancer cell line to the inhibition of metabolic activity after exposure to tested imidazolium salts (Figure 1C). We observed a maximum (to 60%) decrease in metabolic activity of Caco-2 after administering compound **S3**, which caused the strongest depletion of metabolic cells, compared with the control (*p* < 0.001). 

Imidazolium salts, with a combination of satisfactory solubility and high antiproliferative activity, are still actively investigated. Existing research highlights the antitumor properties of some imidazolium salts (IMSs) and their derivatives. Naturally occurring imidazolium salts isolated from a root extract of *Lepidium meyenii* showed cytotoxic activity against various types of human cancer cell lines such as lung carcinoma (A495), colon adenocarcinoma (HT-29), prostate adenocarcinoma (PC3), and kidney carcinoma (A4982LM) [46]. Yang et al. [32] reported a series of imidazolium salts with phenacyl group and their antitumor activities against myeloid leukemia (HL-60 and K562), epidermoid carcinoma (A431), ovarian carcinoma (SKOV-3), gastric carcinoma (MKN-28), liver carcinoma (SMMC-7721), laryngeal carcinoma (Hep-2), and lung carcinoma (GLC-15). A structure–activity relationship study of tested IMSs indicated higher cytotoxic activities for substituted compounds.

Thus far, it has been shown that elongation of the alkyl chain until C-12 increases their cytotoxicity [47,48,49,50]. In their studies with commercially available alkyl imidazolium salts tested on human cancer cell lines, Malthotra et al. showed that IMS with an alkyl chain of C-12 was more effective than compounds with shorter chain lengths [51]. Subsequent studies with the 1-mesityl-3-(2-naphthoylmethano)-1*H*-imidazolium bromide (MNIB) indicated that new imidazolium agents inhibited the growth of different types of tumor cells. Among the tested cell lines, myeloid leukemia (K562), human Burkitt’s lymphoma (Raji), and hepatocellular liver carcinoma (HepG2) exhibited the highest sensitivity to MNIB [16]. Further research with dibenzo [b,d]furane-imidazole hybrid compounds exhibited cytotoxic activity selectively against lung carcinoma (A549) and myeloid liver carcinoma (SMMC-7721) with the G1 phase cell cycle arrest and apoptosis [13]. A series of novel 4-substituted 2,3,6,7-tetrahydrobenzo [1,2-*b*;4,5-*b*′]difuran-1*H*-imidazolium salts also exhibited cytotoxic effect against A549 and SW480 cell lines [38]. De Bord et al. [37] showed high anticancer activity of alkyl- and *N*,*N*′-*bis*naphthyl-substituted imidazolium salts against lung cancer cell lines (NCI–H460, NCI–H1975, HCC827, A549). Further research with a novel steroid imidazolium salt demonstrated the cytotoxic activities of diosgenin–imidazolium derivatives against HL-60, SMMC-7721, A549 MCF-7, and SW480 cell lines [39]. Another imidazolium salt (TPP1), with a triphenylphosphonium substituent, caused apoptotic cell death in bladder cancer cells [52]. A recent study with novel *bis*-imidazolium salts demonstrated the anticancer properties against several non-small-cell lung cancer (NSCLC) lines, where increasing the length of the alkyl chain has been shown to correlate with an increase in antiproliferative activity [41]. As shown in these studies, steroidal imidazolium salt derivatives have a great influence on anticancer potential. The above-mentioned studies indicate the general trend of increased antiproliferative effects of synthesized agents depending on their structure, as well as the presence of substituents and the alkyl chain length.

The cytotoxic effect of IMSs substituted with lithocholic acid on colorectal cancer has not been studied so far. Our in vitro research clearly showed that LCA-based imidazolium salts significantly reduced the viability and metabolic activity of colorectal cancer cells, especially DLD-1 and HT-29 lines, and the anticancer activity of this series of imidazolium salts was directly related to the alkyl chain length, achieving the best results for compounds **S6**–**S8** in DLD-1 and **S3**–**S10** in HT-29.

### 2.2. Effect of IMSs in In Vivo Model

**Figure 2:**
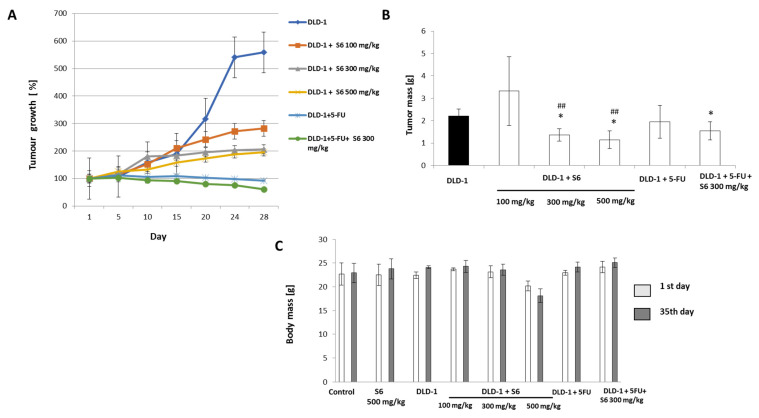
Tumor growth (**A**), tumor mass (**B**), and body mass (**C**) changes in studied groups. The results are expressed as the mean ± SEM for each group. * *p* < 0.05, compared with the DLD-1 group; ## *p* < 0.01 compared with the DLD-1 + **S6** 100 mg/kg.

The comparison of IC50 values (for DLD-1 and CRL1475 cell lines), as well as the results of metabolic activity for DLD-1 **S6** salt, was chosen for further in vivo studies. First, we decided to study the effects of **S6** on healthy animals by administering the highest dose of **S6** (500 mg/kg), to verify that the administered dose will not cause any toxic effects. All mice survived throughout the experiment, and **S6** was well-tolerated in the treated dose regimen. The studies showed that administration of the tested salt **S6** (500 mg/kg), for healthy mice, did not cause significant differences in morphological parameters (Table 2), compared with the control group. Moreover, the administration of **S6** in 100–500 mg/kg doses in animals with DLD-1 tumors did not result in significant morphological changes, compared with the control group. It is worth noting that administration of salt **S6** in the range of tested doses improved RBC, WBC, HGB, and HCT parameters in comparison with the untreated DLD-1 group. In addition, the administration of the tested salt to healthy animals did not impair their condition, measured as a change in body mass during the study. The results (Figure 2C) showed that a decrease in body mass (about 2 g) was noticed only in DLD-1 + 500 mg/kg group after 28 days of **S6** administration. 

The strongest reduction in WBC count was found in the group that received 5-fluorouracil (5-FU) at a dose of 30 mg/kg. Notably, 5-FU is an anticancer chemotherapeutic that also causes a decrease in white and red blood cells, as well as platelet counts [52]. Simultaneous application of **S6** at a dose of 300 mg/kg and 5-FU gave better results in WBC values in comparison to the group that received 5-FU alone. The obtained results unequivocally showed that the use of **S6** was not toxic for the tested animals and even slightly enhanced the blood morphological results of mice treated with 5-FU.

The tumor growth analysis (Figure 2A and Supplementary Data Table S1) showed that the highest tumor volume was observed in the control group (DLD-1). On day 15 of the study, a significantly (*p* < 0.05) slower tumor growth was observed in the groups receiving **S6** (500 mg/kg), 5-FU, and 5-FU with **S6** (300 mg/kg), compared with the control animals. After 24 days, the tumor growth, measured as a percentage increase in tumor volume, was significantly slower in all tested groups than in DLD-1. In the final phase of the experiment, the tumor volume in animals increased to 558%. In the group that received salt **S6**, the tumor volume was significantly lower than that of the control group; specifically, in the final phase of the experiment, it increased to 282.40%, 205.90%, and 196.23% for 100, 300, and 500 mg/kg doses, respectively (Figure 2A). The most significant reduction in tumor mass was noticed in groups with DLD-1 that received **S6** at dosages of 300 and 500 mg/kg (Figure 2B). We found a reduction in tumor growth (the tumor volume decreased to 93.27%) in the group with 5-FU. The strongest inhibition of tumor growth (a decrease in tumor volume of 61.47%) and tumor mass of about 31% was observed after the simultaneous administration of test salt **S6** at a dose of 300 mg/kg with 5-FU. This clearly indicated that tested imidazolium salt **S6** slowed tumor growth in a dose-dependent manner. These results revealed the beneficial interaction of imidazolium salt **S6** and 5-FU in inhibiting CRC tumor growth.

**Figure 3:**
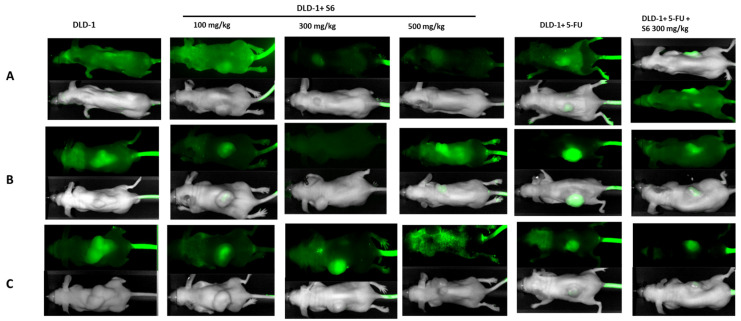
Imaging of the tumor sizes during administration of **S6** for 28 days in the study groups on the 1st (**A**), 14th (**B**), and 28th (**C**) day using the Pearl system.

Moreover, intravital imaging of mice was performed using the Pearl system on the 7th, 14th, and 28th day of the experiment (Figure 3). The presented images show that the fastest tumor growth was observed in the DLD-1 group, while administration of **S6** or 5-FU indeed resulted in slower tumor growth. The strongest decrease in tumor volume was noticed in the group that received **S6** at a dose of 300 mg/kg and 5-FU.

Our results have shown that tested imidazolium salt **S6** reduces the growth of induced colorectal tumor, whereas in combination with a chemotherapeutic agent, it provides the most beneficial effect in inhibiting CRC tumor growth. Haque et al. [52] tested Ag(I)-N-heterocyclic carbene complexes of N-allyl substituted imidazol-2-ylidenes against HCT 116 cell line and showed high cytotoxic activity which was significantly higher compared to the standard drug 5-FU. Moreover, the HCT116 cell line xenograft mouse model demonstrated that infliximab (anti-tumor necrosis factor α monoclonal antibody) and 5-FU combination significantly inhibited tumor growth compared to the alone 5-FU treatment [53]. Our results also confirm a better therapeutical effect of 5-FU combined with imidazolium salts in CRC treatment. 

The first animal model of antitumor properties of IMSs was tested on human non-small lung tumor (A549) xenografts [16]. The tumor volumes were significantly inhibited after 19 days of 1-mesityl-3-(2-naphthoylmethano)-1*H*-imidazolium bromide (MNIB) intraperitoneally administration in doses 4 and 8 mg/kg (*p* < 0.05–0.001) compared to the control group. Moreover, after application of MNIB in the range of 10–80 mg/kg no evidence of acute toxicity was found. In another study, a Huh7 hepatocellular carcinoma xenograft mouse model that received water containing IBN-1 (1,3-*bis*benzylimidazolium bromide, 2 g/L) or IBN-9 (1-mesityl-3-(4-acetate-benzyl bromide, 0.6 and 1.5 g/L) [33]. After three weeks in a group with IBN-1, a reduction of tumor volume by 31% was observed, but it was accompanied by a 9% decrease in body mass. By comparison, IBN-9 significantly reduced the tumor volume by 45% and 60% at a dose of 0.6 and 1.5 g/L, respectively, without loss of body weight. In another study *N*,*N*′-*bis*(arylmethyl)imidazolium salt, with anticancer activities, was tested in vivo in toxicity study [36]. All animals survived but there was a decrease in weight after the first injection of a tested agent. However, this was followed by complete recovery and 66% of the animals had gained weight by day six. Further five injectionsadministered during 30 days resulted in a similar model of body weight loss and gain. Stromyer et al. [40] applied intravesically to a bladder in a cancer mouse model an imidazolium salt with triphenylphosphonium substituent (TPP1). The study showed that TPP1 caused necrosis and apoptosis by the cleavage of caspase-3 in cancer cells without damaging normal cells in the bladder. The recent research with IMSs also showed well tolerance of *bis*-imidazolium salts in vivo [41]. Injection in C57BL/6 mice of tested salt in dose 20 mg/kg by 30 days caused an average weight loss of 4% and all animals survived for the entire duration of the experiment. 

The presented studies showed anticancer activities and low toxicity of imidazolium salts in in vivo model. Our research demonstrated that imidazolium salt substituted with lithocholic acid and hexyl chain (**S6**) in doses 100–500 mg/kg contributed to the slower growth of colorectal cancer tumors in an animal model without toxic health effects. In addition, we have shown that the tested salt in combination with 5-FU has better results in inhibiting colorectal tumor growth compared with 5-FU alone. Taken together, LCA-based imidazolium salts treatment can enhance the therapeutic effects of colon cancer chemotherapy.

### 2.3. Effect of IMSs on Biochemical Parameters

**Figure 4:**
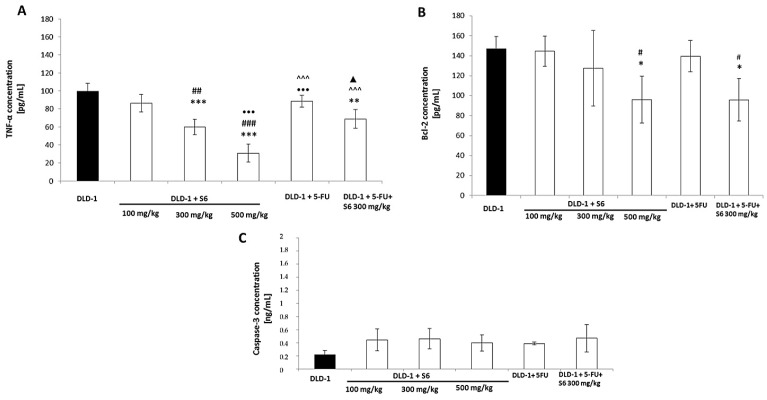
TNF-α (**A**), Bcl-2 (**B**), and caspase-3 (**C**) concentrations in the serum of mice from experimental groups. The results are expressed as the mean ± SEM for each group. * *p* <0.05, ** *p* < 0.01 and *** *p* < 0.001 compared with the DLD-1 group; # *p* < 0.05, ## *p* < 0.01, and ### *p* < 0.001 compared with DLD-1 + **S6** 100 mg/kg; ••• *p* < 0.001 compared with DLD-1+ **S6** 300 mg/kg; ^^^ *p* < 0.001 compared with DLD-1 + **S6** 500 mg/kg; ▲ *p* < 0.05 compared with DLD-1+ 5FU.

Experimental and clinical studies demonstrated that tumor necrosis TNF-α plays a key role in the progression of colon cancer [54,55,56,57]. TNF-α is a pro-inflammatory cytokine released by monocytes as well as tumor cells that intensifies oncogenic activation, cancer cell invasion, and angiogenesis [57]. Proneovascular activity of TNF-α may be associated with the activation by this pro-inflammatory cytokine of enzymes that promote the spread of cancer cells, or increased adhesion of cancer cells to the vascular endothelium. TNF-α levels in serum patients with prostate or colon cancer were reduced after the application of chemotherapy, which would suggest the usefulness of TNF-α determination as a marker of response to the applied anticancer treatment [54,57]. We decided to examine the concentration of TNF-α in the serum of tested mice. The results showed the differences in its level in tested groups (Figure 4A). The highest concentration was noticed in the DLD-1 group (99.6 pg/mL), while in the DLD-1+500 mg/kg **S6** the lowest (30.9 pg/mL) level of TNF-α was found. A significant reduction of TNF-α level compared to the DLD-1 group was also observed in mice receiving **S6** of 300 mg/kg (*p* < 0.001). As presented, 5-FU treatment did not cause a significant decrease of TNF-α level, compared to the DLD-1 group. The combination of 5-FU and **S6** (300 mg/kg) showed a reduction of TNF-α concentration compared to the **S6** (100 mg/kg) (statistically insignificant) which was not found in comparison with **S6** (300–500 mg/kg). A 5-FU with **S6** (300 mg/kg) showed a reduction of TNF-α compared with DLD-1 + 5-FU (statistically insignificant) and a significant decrease compared to the DLD-1 group (*p* < 0.01). The combination of imidazolium salt **S6** with 5-FU showed a better response in vivo than 5-FU alone.

High TNF-α level induces a range of downstream pathways such as the NF-κB pathway. NF-κB activation induces negative regulators of apoptosis such as Bcl-2 [58]. The expression of TNF-α in tumors is responsible for the activation of NF-κB and thus promotes apoptosis resistance. Bcl-2 concentrations were also determined in our tests. The proteins of Bcl-2 and caspase-3 are associated with cell apoptosis. Bcl-2 is a member of important antiapoptotic proteins that inhibits cell apoptosis, while caspase-3 belongs to a cysteine protease family, which plays a crucial role in apoptotic pathways. Taking the essential role of caspase-3 in apoptosis into account, we examined the level of caspase-3 in the serum of mice with DLD-1 cells that received **S6**. The results showed the differences in the concentration of Bcl-2 and caspase-3 in the tested groups (Figure 4B). The highest level of Bcl-2 was noticed in the DLD-1 group (146.9 pg/mL). The findings revealed that the application of tested imidazolium salt **S6** (100–500 mg/kg) caused a dose-dependent decrease in Bcl-2 concentration in the tested groups: DLD-1 + 100 mg/kg (144.6 pg/mL); DLD-1 + 300 mg/kg (127.5 pg/mL); and DLD-1 + 500 mg/kg (96.0 pg/mL). There were no significant differences in Bcl-2 concentrations between mice with 5-FU (139.5 pg/mL) and the control group. However, it is worth noting that the level of Bcl-2 in the group with 5-FU and imidazolium salt **S6** (300 mg/kg) significantly decreased, compared with the control (*p* < 0.05) and mice treated with 5-FU. These results indicated that the tested salt **S6** decreased Bcl-2 concentration in the serum of mice with DLD-1 cells. 

Based on our results, there were no statistically significant differences in the case of caspase-3 concentration between the tested group (Figure 4C). In the control group (with DLD-1), we noted the lowest concentration of caspase-3 (0.218 ng/mL). The results showed an increasing trend in caspase-3 levels in groups with imidazolium salt (100–500 mg/kg), compared with the control. It should be pointed out that the concentration of caspase-3 in the group with 5-FU and imidazolium salt **S6** (300 mg/kg) was accordingly higher (0.468 ng/mL) than those receiving 5-FU alone (0.388 ng/mL). Moreover, the level of caspase-3 in the group with imidazolium salt and 5-FU was comparable to the result in a group with **S6** at a dose of 300 mg/kg (0.461 ng/mL). These results suggest that tested imidazolium salt increases the concentration of caspase-3 and induces apoptosis in animals with DLD-1 cells.

The recent in vivo studies with the bladder cancer mouse model showed that mice treated with an imidazolium salt including a triphenylphosphonium substituent (TPP1) had a higher level of cleaved caspase-3 in the tissues that had a significant amount of necrosis [38].

### 2.4. Effect of IMSs on Histopathological Analysis

**Figure 5:**
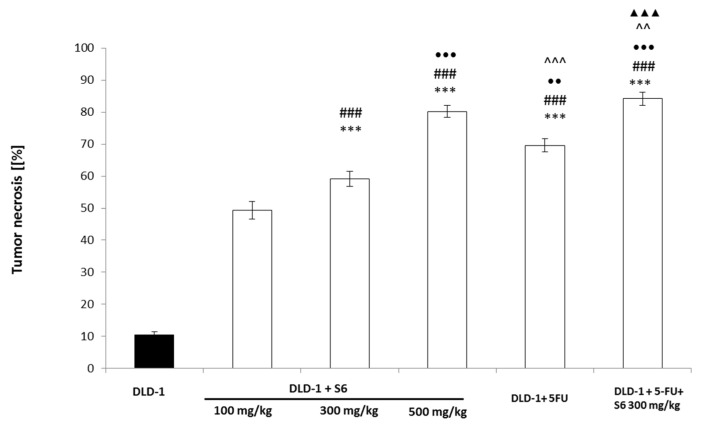
Tumor necrosis in induced CRC tumors from experimental groups. The results are expressed as the mean ± SEM for each group. *** *p* < 0.001 compared with the DLD-1 group; ### *p* < 0.001 compared with the DLD-1 + **S6** 100 mg/kg; •• *p* < 0.01 and ••• *p* < 0.001 compared with the DLD-1 + **S6** 300 mg/kg group; ^^*p* < 0, 01 and ^^^ *p* < 0.001 compared with the DLD-1 + **S6** 500 mg/kg group; ▲▲▲ *p* < 0.001 compared with the DLD + 5-FU.

**Figure 6:**
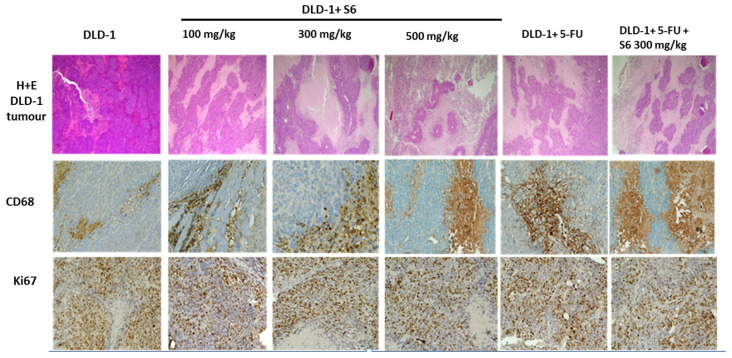
H&E staining, CD68, and Ki67 immunohistochemical expressions for DLD-1 tumor **(200× magnification)**.

The histopathological analysis showed a significant increase in tumor necrosis in the tested groups, compared with the control (Figure 5 and Figure 6). The degree of tumor necrosis increased in a concentration-dependent manner in groups that received the tested salt **S6**, reaching 80.2% of tumor necrosis in animals administered the tested agent at a concentration of 500 mg/kg. In the group that received 5-FU, we found 69.6% tumor necrosis, whereas the use of 5-FU and imidazolium salt at a concentration of 300 mg/kg noticeably resulted in the highest effect (84.23%).

In our studies, we assessed the presence of macrophages with CD68 expression in tumors of tested groups. The immunohistochemical analysis revealed differences in CD68 levels—namely, the number of macrophages increased with the concentration of tested imidazolium salt **S6** (Table 3 and Figure 6). The highest number of macrophages was observed in tumors collected from animals in the following groups: DLD-1 + 500 mg/kg, DLD-1 + 5FU, and DLD-1 + 5FU + 100 mg/kg. We also observed that macrophage numbers increased the level of necrosis in induced CRC tumors. Growing evidence suggests that the tumor microenvironment (TME) plays an important role in cancer progression and development. TME is a unique environment that develops alongside tumor progression. The immune microenvironment significantly affects tumor development. Among the immune cells recruited to the tumor site, tumor-associated macrophages (TAMs) are the most abundant at all stages of tumor progression. They play important roles by secreting cytokines and chemokines and coordinating with inflammatory mechanisms to promote tumor progression and drug tolerance. Some studies have shown that CD68 TAM in CRC tissues was significantly associated with better overall survival (OS) rates [59]. Moreover, studies presented by Edin et al. showed that high macrophage infiltration is associated with a better prognostic in CRC patients in a stage-dependent manner [60].

In the studied tumors, we also determined the expression of Ki67, which is strictly associated with cell proliferation. Ki67 protein is present in all active phases of the cell cycle (G1, S, G2, and mitosis); therefore, it is the best marker to determine the growth of cell population, and likewise, it can indicate growing tumor cells. The results showed that the expression of Ki67 in the DLD-1 group was about 80%, whereas, in all groups treated with chemotherapeutic agents (**S6** or 5-FU), it was similar and was estimated to be about 60% (Table 3 and Figure 6). DLD-1 is a colorectal adenocarcinoma cell line, Dukes’ type C, which is characterized by a high degree of proliferation, thus indicating a high degree of malignancy of this tumor. A study presented by Melling et al. [59], in which Ki67 expression was analyzed on 1800 CRC samples, showed that a low Ki67 index is associated with low tumor stage and is an independent prognostic factor of favorable survival. Forones et al. [61] hypothesized that Ki67 is not correlated with clinical and pathologic parameters. Through a series of in vivo studies, derivatives of imidazolium salt (IBN-9) in a hepatocellular carcinoma mouse model were tested. The results showed that IBN-9 (2 g/L) decreased the protein expression of Ki67 to 66% and also caused necrosis in tumor tissues [33]. Recent in vivo studies with a bladder cancer mouse model showed that mice treated with a triphenylphosphonium-containing imidazolium salt (TPP1) had higher levels of cleaved caspase-3 in the tissues that had significant amounts of necrosis [38]. 

Our results showed that tested imidazolium salt **S6** caused an increase in the number of macrophages that induce tumor breakdown and necrosis, resulting in better therapeutic effects of CRC growth inhibition.

## 3. Materials and Methods

### 3.1. Synthesis of Imidazolium Salts

**Figure 7:**
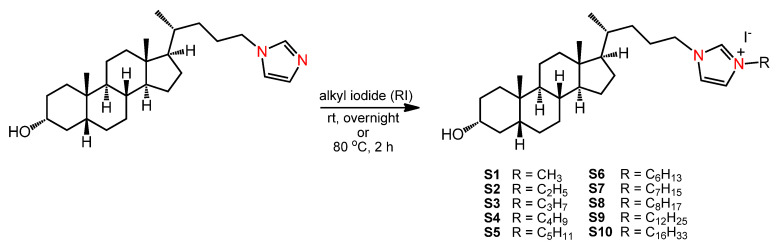
Synthesis of imidazolium salts based on lithocholic acid from *N*-LCA-substituted imidazole.

We recently described the synthesis of imidazolium salts based on lithocholic acid with substituents of different alkyl chain lengths [29,31]. New salts are almost universal and are broad-spectrum, antibacterial, and antifungal agents. Two salts with the shortest chain length (methyl and ethyl) were prepared from *N*-LCA-substituted imidazole according to the first reported procedure [31]. The remaining salts with longer side chains were prepared via the modified synthesis, which allowed to shorten the reaction time and obtain compounds with higher yields [31] (Figure 7). As a result, ten salts (**S1**–**S10**) of different chain lengths, depending on the alkyl iodide used, were obtained to be subjected to anticancer tests.

### 3.2. Cell Culture

The anticancer activities of imidazolium salts (**S1**–**S10**) were evaluated against colon cancer cell lines (DLD-1, HT-29, Caco-2) originating from American Type Culture Collection (Manassas, VA, USA). Cells were grown in 96-well plates at 5–7 × 10^3^ cells per well to full confluence in RPMI-1640 (ATCC) (for DLD-1 line), in McCoy’s 5A (ATCC) (for HT-29 line), and in Eagle’s minimum essential medium (EMEM) (for Caco-2 line) supplemented with 10% fetal bovine serum (FBS) (Eurx, Gdańsk, Poland), 50 U/mL penicillin, and 50 mg/mL streptomycin (Gibco, Thermo Fisher Scientific, Inc., Waltham, MA, USA) under physiological conditions, at 37 °C with 5% CO_2_, and passed every 2–3 days. Cells used in assays originated from 6–14 passages.

### 3.3. Cytotoxicity Assay (IC50 Values)

To evaluate the cytotoxicity of tested salts (**S1**–**S10**), a neutral red cell cytotoxicity assay was used. The principle of this assay is based on the detection of viable cells via the uptake of the dye-neutral red. Neutral red is a eurhodin dye that stains lysosomes in viable cells. Viable cells can take up neutral red via active transport and incorporate the dye into their lysosomes, but nonviable cells cannot take up this chromophore. After 24 h incubation of cells with tested salts (1, 5, 10, 30, 50, and 100 µg/mL), a neutral red solution (7 µL/well) was added and incubated for 2 h. Afterward, the culturing medium was removed, and the cells were fixed for 5 min. A fixative solution was discarded, and then the acidic solution was added to dissolve the dye. The absorbance was measured at a wavelength of 540 nm on a BioTek Epoch plate reader [62]. The IC50 values were calculated using nonlinear regression (three parameters) in GraphPad Prism.

### 3.4. Metabolic Activity of Cells

The metabolic activity of colon cancer cells after the addition of tested salts (**S1**–**S10**) was determined via the MTT assay method [63]. All salts were dissolved in PBS and diluted in fresh media to the desired concentrations (1, 5, 10, 30, 50, and 100 µg/mL). After 24 h of cells incubation with tested salts, the MTT assay protocol was followed. The culturing medium was discarded, and the cells were rinsed with PBS three times. Then, MTT reagent (5 mg/mL) was added to each well and incubated for 0.5–1 h depending on the cell line. The medium was removed from the wells and 100 µL of DMSO (Alchem, Poland) with 10 µL of Sorren’s buffer (0.1 mol/L glycine with 0.1 mol/L NaCl equilibrated to pH 10.5) was added. The optical density was recorded with a BioTek Epoch plate reader spectrophotometer at a wavelength of 570 nm. Values are described as a percent of control.

### 3.5. In Vivo Evaluation of Salt **S6**

All procedures were carried out in accordance with the European Directive (2010/63/EU). Handling of animals was performed with the consent of protocols approved by the Local Ethical Committee in Olsztyn no 74/2019.

### 3.6. DLD-1 Xenograft Model

Nude male mice (Cby.Cg.Foxn1<nu>/cmdb), 5–6 weeks old, were housed under controlled conditions (21 ± 2 °C, 12 h light/12 h dark cycle) with unlimited access to water. DLD-1 cells (2 × 10^7^) in 0.1 mL volume of Matrigel/PBS (Alchem, Poland) mix were implanted subcutaneously into the flanks of nude mice. After 7 days, mice were randomly divided into six groups (*n* = 6), which were given intragastrically once a day for 28 days PBS (control group), salt **S6** (100, 300, and 500 mg/kg b.w.), salt **S6** (300 mg/kg b.w.) with an intraperitoneal injection of 5-fluorouracil (30 mg/kg), and group with 5-fluorouracil. The 5-fluorouracil injection was given on the 14th, 21st, and 28th day of the experiment. The tumor volume was measured twice a week (every 3–4 days) via a digital caliper and calculated using the following formula: (0.52 × width^2^ × length). Tumor growth was shown as a percentage increase in volume to baseline, which was treated as 100%. In addition, in order to illustrate the growth rate of the tumor, mice were given intravenously IRDye 800CW 2-deoxyglucose (100 µL) and after 24 h using the Pearl system on the 7th, 14th, and 28th day of the experiment. After 35 days, mice were sacrificed, and blood and organs were taken for further analysis. The morphological examination of blood collected from the animals was performed using the ABCvet system.

For the initial assessment of the impact of potential adverse reactions of salt **S6**, an additional group of mice (*n* = 6) received the dose intragastrically once a day for 28 days (500 mg/kg b.w.). After 28 days, mice were sacrificed, and blood and organs were taken for further analysis.

### 3.7. Biochemical Analysis

The biochemical assays (enzyme-linked immunosorbent assays) were made in the serum collected from all mice with DLD-1 cells. TNF-α, Bcl-2, and caspase-3 concentrations were determined using the commercial Enzyme-linked Immunosorbent Assay Kit for mice (Cloud-Clone Corp. Unit, Katy, TX, USA). The absorbance was measured in a microtiter plate reader the Epoch (BioTek). All results were performed in duplicate samples.

### 3.8. Histopathological and Immunohistochemical Analysis

Tumor tissue sections were taken during the surgery, fixed in buffered formalin solution, and embedded in paraffin. The paraffin blocks were sliced with a microtome into 4 μm thick sections, placed on silanized slides, and stained with hematoxylin and eosin. The histopathological examination evaluated the percentage of necrotic area in relation to tumor area that was calculated using an Olympus EP50 camera. For immunohistochemical analysis, the slides were incubated overnight at 60 °C and then deparaffinized in xylene solutions and rehydrated in a series of alcohols in concentrations of 99.9%, 96%, and 70%. Next, the sections were placed in citrate buffer (pH = 6.0) and incubated in a water bath for 20 min at 98.5 °C, to reveal the antigen, and then incubated for 20 min at room temperature. Endogenous peroxidase was blocked by using 3% hydrogen peroxide for 10 min, followed by 2.5% goat serum, to block nonspecific antibody binding (Vector Laboratories, Eching, Germany) for 10 min. Then, the slides were incubated with specific antibodies—Ki67 (Biorbyt, dilution 1:100) and CD68 (Biorbyt, dilution 1:100)—for 30 min at room temperature. Then, antibody-binding sites were visualized using the ImmPress Goat Anti-Rabbit IgG Polymer Reagent kit (Vector Laboratories, Eching, Germany) for 30 min and ImmPACT DAB chromogen (Vector Laboratories, Eching, Germany) for 5 min. Cell nuclei were stained with hematoxylin, and then the preparations were dehydrated in a series of alcohols with increasing concentrations of 70%, 96%, and 99.9%, and washed in xylene solutions. The evaluation of immunohistochemical staining was performed with a light microscope using 200× magnification. For each immunohistochemical staining were performed measurements of immunohistochemical reaction intensity per group of mice.

### 3.9. Statistical Analysis

All results are expressed as mean ± SD. Statistical analysis was performed using Statistica 13.3 version (StatSoft, Tulsa, OK, USA). Intergroup statistical comparisons were analyzed using standard statistical analyses, including one-way analysis of variance (ANOVA), followed by post hoc Duncan’s or Tukey’s comparison test. Differences were considered significant when *p* < 0.05.

## 4. Conclusions

In the presented research, for the first time, the cytotoxic effect against CRC of the series of novel imidazolium salts substituted with lithocholic acid was demonstrated.

Our in vitro research clearly showed that LCA-based imidazolium salts significantly reduced the viability and metabolic activity of colorectal cancer cells, especially in DLD-1 and HT-29 lines, and that the anticancer activity of these series of imidazolium salts was directly related to the alkyl chain length, which achieved the best results for compounds **S6**–**S8** for DLD-1 and **S4, S8**–**S10** for HT-29. In vivo studies showed low toxicity of **S6** to healthy animals and inhibition of tumor growth in the xenograft model of colon cancer, with better results than those obtained with 5-FU. Furthermore, the obtained results showed that administration of LCA-IMSs decreased TNF-α and Bcl-2 concentrations, with a significant amount of necrosis in DLD-1 tumors.

The next step will be to extend the studies on different cell lines and develop further schemes in in vivo models to confirm the demonstration of antitumor effects of lithocholic acid-based imidazolium salts, highlighting their application in adjuvant anticancer therapy.

## Figures and Tables

**Table 1:** Cytotoxic activity of imidazolium salts (**S1**–**S10**) in vitro (IC50).

Compound (IMS)	*N*-Substituent	Colon Cancer Cell Line						Fibroblast Cells CRL-1475 [31]	
		DLD-1		HT-29		Caco-2			
		µg/mL	µM/mL	µg/mL	µM/mL	µg/mL	µM/mL	µg/mL	µM/mL
**S1**	*methyl*	113.4 ± 0.10	0.20 ± 0.05	130.3 ± 0.12	0.23 ± 0.02	348.5± 0.21	0.63 ± 0.04	107.4 ± 2.34	0.19 ± 0.01
**S2**	*ethyl*	116.4 ± 0.17	0.20± 0.01	146.1 ± 0.34	0.26 ± 0.04	380.3± 1.03	0.67 ± 0.02	113.9 ± 1.78	0.20 ± 0.02
**S3**	*propyl*	109.5 ± 0.19	0.19 ± 0.03	88.4 ± 0.59	0.15 ± 0.03	401.6± 1.34	0.69 ± 0.01	36.5 ± 0.76	0.06 ± 0.01
**S4**	*butyl*	154.3 ± 0.43	0.26 ± 0.03	66.6 ± 0.75	0.11 ± 0.02	661.4± 1.95	1.11 ± 0.03	99.2 ± 1.12	0.17 ± 0.02
**S5**	*pentyl*	142.6 ± 0.24	0.23 ± 0.01	103.6 ± 1.34	0.17 ± 0.01	959.0± 1.56	1.57 ± 0.04	100.8 ± 2.76	0.17 ± 0.01
**S6**	*hexyl*	78.9 ± 0.21	0.13 ± 0.04	101.3 ± 0.95	0.16 ± 0.02	250.1± 0.31	0.40 ± 0.06	149.6 ± 3.01	0.24 ± 0.03
**S7**	*heptyl*	98.3 ± 0.22	0.15 ± 0.01	92.5 ± 1.13	0.14 ± 0.01	84.5± 0.89	0.13 ± 0.01	134.4 ± 4.17	0.21 ± 0.02
**S8**	*octyl*	79.8 ± 0.24	0.12 ± 0.03	73.9 ± 0.47	0.11 ± 0.02	131.2± 1.54	0.20 ± 0.01	53.6 ± 0.57	0.08 ± 0.01
**S9**	*dodecyl*	132.2 ± 0.64	0.19 ± 0.02	76.5 ± 0.67	0.11 ± 0.01	723.6 ± 0.76	1.02 ± 0.02	631.0 ± 4.78	0.89 ± 0.06
**S10**	*hexadecyl*	151.9 ± 0.98	0.20 ± 0.02	80.6 ± 0.34	0.11± 0.01	188.0± 0.72	0.25 ± 0.01	841.1 ± 4.76	1.10 ± 0.03

**Table 2:** Morphological blood parameters of control and experimental mice.

Parameters	Control	S6 500 mg/kg	DLD-1	DLD-1 + S6			DLD-1 + 5-FU 30 mg/kg	DLD-1 + 5-FU + S6 300 mg/kg
				100 mg/kg	300 mg/kg	500 mg/kg		
**WBC (10^3^/mm^3^)**	2.53 ± 0.45	2.33 ± 0.31	1.83 ± 0.55	2.60 ± 0.57	2.15 ± 0.59	1.93 ± 0.31	1.28 ± 0.76 ^a,b^	1.76 ± 0.34 ^e^
**RBC (10^6^/mm^3^)**	10.01 ± 0.03	9.91 ± 0.45	8.0 ± 2.40	9.95 ± 0.20	10.38 ± 0.38	10.07 ± 0.69	10.67 ± 0.66 ^b^	10.48 ± 0.38 ^c^
**HGB (g/dl)**	14.53 ± 0.12	15.05 ± 0.65	13.63 ± 2.16	14.60 ± 0.42	15.36 ± 0.57	14.53 ± 1.08	15.43 ± 1.07	15.36 ± 0.69
**HCT (%)**	53.43 ± 0.67	53.72 ± 2.38	50.43 ± 9.24	54.55 ± 1.77	52.29 ± 9.42	53.98 ± 4.97	57.18 ± 3.97	57.04 ± 2.71
**MCV (µm^3^)**	53.33 ± 0.58	54.17 ± 0.75	54.25 ± 0.50	54.50 ± 0.71	53.82 ± 1.74	53.75 ± 1.50	53.25 ± 0.5	54.40 ± 0.89
**MCH (pg)**	14.50 ± 0.17	15.20 ± 0.25	14.80 ± 0.43	14.65 ± 0.07	18.81 ± 2.71	14.40 ± 0.14	14.48 ± 0.10	14.68 ± 0.23
**MCHC (g/Dl)**	27.37 ± 0.12	28.02 ± 0.25	27.18 ± 0.92	26.75 ± 0.21	26.35 ± 4.12	26.88 ± 0.55	26.98 ± 0.29	26.92 ± 0.20
**PLT (10^3^/mm^3^)**	731.33 ± 98.43	598.83 ± 201.32	668.50 ± 346.91	497.50 ± 89.80	674.67 ± 123.57	275.25 ± 77.62 ^a,b,c^	704.50 ± 141.06 ^d^	681.00 ± 231.92 ^e^

The results are presented as the mean values + SD. **^a^** *p* < 0.05 vs. the control; **^b^** *p* < 0.05 vs. DLD-1 + 100 mg/kg; **^c^** *p* < 0,05 vs. DLD-1 + 300 mg/kg; **^d^** *p* < 0,05 vs. DLD-1 + 500 mg/kg; **^e^** *p* < 0,05 vs. DLD-1 + 5-FU. Abbreviations: WBC, white blood cells; RBC, red blood cells; HGB, hemoglobin; HCT, hematocrit; MCV, mean corpuscular volume; MCH, mean corpuscular hemoglobin; MCHC, corpuscular/cellular hemoglobin concentration; PLT, platelets.

**Table 3:** Expression of CD68 and Ki67 in the DLD-1 tumor.

	Expression	
	CD68	Ki67
DLD-1	+	+++
DLD-1 + 100 mg/kg	++	++
DLD-1 + 300 mg/kg	++	++
DLD-1 + 500 mg/kg	+++	++
DLD-1 + 5FU	+++	++
DLD-1+ 5FU + 500 mg/kg	+++	++

(+) when observed in <20% of cells; (++) 21–60% of cells; (+++) >61% of cells.

## Data Availability

Not applicable.

## Supplementary Materials

The following supporting information can be downloaded at: https://www.mdpi.com/article/10.3390/ijms23137019/s1.

## References

[1] Alyabsi M., Algarni M., Alshammari K. (2021). Trends in Colorectal Cancer Incidence Rates in Saudi Arabia (2001–2016) Using Saudi National Registry: Early- versus Late-Onset Disease. Front. Oncol..

[2] Hull R., Francies F.Z., Oyomno M., Dlamini Z. (2020). Colorectal Cancer Genetics, Incidence and Risk Factors: In Search for Targeted Therapies. Cancer Manag. Res..

[3] Siegel R.L., Miller K.D., Goding Sauer A., Fedewa S.A., Butterly L.F., Anderson J.C., Cercek A., Smith R.A., Jemal A. (2020). Colorectal Cancer Statistics, 2020. CA Cancer J. Clin..

[4] Quirke P., Steele R., Monson J., Grieve R., Khanna S., Couture J., O’Callaghan C., Myint A.S., Bessell E., Thompson L.C. (2009). Effect of the Plane of Surgery Achieved on Local Recurrence in Patients with Operable Rectal Cancer: A Prospective Study Using Data from the MRC CR07 and NCIC-CTG CO16 Randomised Clinical Trial. Lancet.

[5] Brown S., Banfill K., Aznar M.C., Whitehurst P., Faivre Finn C. (2019). The Evolving Role of Radiotherapy in Non-Small Cell Lung Cancer. Br. J. Radiol..

[6] Sauer R., Becker H., Hohenberger W., Rödel C., Wittekind C., Fietkau R., Martus P., Tschmelitsch J., Hager E., Hess C.F. (2004). Preoperative versus Postoperative Chemoradiotherapy for Rectal Cancer. N. Engl. J. Med..

[7] Glimelius B., Tiret E., Cervantes A., Arnold D. (2013). Rectal Cancer: ESMO Clinical Practice Guidelines for Diagnosis, Treatment and Follow-Up. Ann. Oncol..

[8] Riduan S.N., Zhang Y. (2013). Imidazolium Salts and Their Polymeric Materials for Biological Applications. Chem. Soc. Rev..

[9] Zhang Y., Chan J.Y.G. (2010). Sustainable Chemistry: Imidazolium Salts in Biomass Conversion and CO_2_ Fixation. Energy Environ. Sci..

[10] Borowiecki P., Milner-Krawczyk M., Brzezińska D., Wielechowska M., Plenkiewicz J. (2013). Synthesis and Antimicrobial Activity of Imidazolium and Triazolium Chiral Ionic Liquids: Synthesis and Antimicrobial Activity of Chiral Ionic Liquids. Eur. J. Org. Chem..

[11] Coleman D., Špulák M., Garcia M.T., Gathergood N. (2012). Antimicrobial Toxicity Studies of Ionic Liquids Leading to a ‘Hit’ MRSA Selective Antibacterial Imidazolium Salt. Green Chem..

[12] Chen Q.-L., Chen H.-B., Cao Z.-X., Zhou Z.-H. (2013). Synthesis, Spectral, and Structural Characterizations of Imidazole Oxalato Molybdenum(IV/V/VI) Complexes. Dalton Trans..

[13] Liu L., Wu H., Riduan S.N., Ying J.Y., Zhang Y. (2013). Short Imidazolium Chains Effectively Clear Fungal Biofilm in Keratitis Treatment. Biomaterials.

[14] Raghavan S., Manogaran P., Gadepalli Narasimha K.K., Kalpattu Kuppusami B., Mariyappan P., Gopalakrishnan A., Venkatraman G. (2015). Synthesis and Anticancer Activity of Novel Curcumin–Quinolone Hybrids. Bioorg. Med. Chem. Lett..

[15] Coa J.C., Castrillón W., Cardona W., Carda M., Ospina V., Muñoz J.A., Vélez I.D., Robledo S.M. (2015). Synthesis, Leishmanicidal, Trypanocidal and Cytotoxic Activity of Quinoline-Hydrazone Hybrids. Eur. J. Med. Chem..

[16] Zeng X., Yang X., Zhang Y., Qing C., Zhang H. (2010). Synthesis and Antitumor Activity of 1-Mesityl-3-(2-Naphthoylmethano)-1H-Imidazolium Bromide. Bioorg. Med. Chem. Lett..

[17] Song W.-J., Yang X.-D., Zeng X.-H., Xu X.-L., Zhang G.-L., Zhang H.-B. (2012). Synthesis and Cytotoxic Activities of Novel Hybrid Compounds of Imidazole Scaffold-Based 2-Substituted Benzofurans. RSC Adv..

[18] Kaushik N., Attri P., Kaushik N., Choi E. (2012). Synthesis and Antiproliferative Activity of Ammonium and Imidazolium Ionic Liquids against T98G Brain Cancer Cells. Molecules.

[19] Chang K.-H., Lee L., Chen J., Li W.-S. (2006). Lithocholic Acid Analogues, New and Potent α-2,3-Sialyltransferase Inhibitors. Chem. Commun..

[20] Benis K.A., Schneider G.B. (1996). The Effects of Vitamin D Binding Protein-Macrophage Activating Factor and Colony-Stimulating Factor-1 on Hematopoietic Cells in Normal and Osteopetrotic Rats. Blood.

[21] Trah J., Arand J., Oh J., Pagerols-Raluy L., Trochimiuk M., Appl B., Heidelbach H., Vincent D., Saleem M.A., Reinshagen K. (2020). Lithocholic Bile Acid Induces Apoptosis in Human Nephroblastoma Cells: A Non-Selective Treatment Option. Sci. Rep..

[22] Goldberg A.A., Beach A., Davies G.F., Harkness T.A.A., LeBlanc A., Titorenko V.I. (2011). Lithocholic Bile Acid Selectively Kills Neuroblastoma Cells, While Sparing Normal Neuronal Cells. Oncotarget.

[23] Luu T.H., Bard J.-M., Carbonnelle D., Chaillou C., Huvelin J.-M., Bobin-Dubigeon C., Nazih H. (2018). Lithocholic Bile Acid Inhibits Lipogenesis and Induces Apoptosis in Breast Cancer Cells. Cell. Oncol..

[24] Dusso A.S., Brown A.J., Slatopolsky E. (2005). Vitamin D. Am. J. Physiol. Renal Physiol..

[25] Mantell D.J., Owens P.E., Bundred N.J., Mawer E.B., Canfield A.E. (2000). 1α,25-Dihydroxyvitamin D3 Inhibits Angiogenesis In Vitro and In Vivo. Circ. Res..

[26] Peters U., McGlynn K.A., Chatterjee N., Gunter E., Garcia-Closas M., Rothman N., Sinha R. (2001). Vitamin D, Calcium, and Vitamin D Receptor Polymorphism in Colorectal Adenomas. Cancer Epidemiol. Biomarkers Prev..

[27] Thanikachalam K., Khan G. (2019). Colorectal Cancer and Nutrition. Nutrients.

[28] Lajczak-McGinley N.K., Porru E., Fallon C.M., Smyth J., Curley C., McCarron P.A., Tambuwala M.M., Roda A., Keely S.J. (2020). The Secondary Bile Acids, Ursodeoxycholic Acid and Lithocholic Acid, Protect against Intestinal Inflammation by Inhibition of Epithelial Apoptosis. Physiol. Rep..

[29] Hryniewicka A., Malinowska M., Hauschild T., Pieczul K., Morzycki J.W. (2019). Synthesis and Antimicrobial Properties of Steroid-Based Imidazolium Salts. J. Steroid Biochem. Mol..

[30] Hryniewicka A., Niemirowicz-Laskowska K., Wielgat P., Car H., Hauschild T., Morzycki J.W. (2021). Dehydroepiandrosterone Derived Imidazolium Salts and Their Antimicrobial Efficacy. Bioorg. Chem..

[31] Malinowska M., Sawicka D., Niemirowicz-Laskowska K., Wielgat P., Car H., Hauschild T., Hryniewicka A. (2021). Steroid-Functionalized Imidazolium Salts with an Extended Spectrum of Antifungal and Antibacterial Activity. Int. J. Mol. Sci..

[32] Yang X.-D., Zeng X.-H., Zhang Y.-L., Qing C., Song W.-J., Li L., Zhang H.-B. (2009). Synthesis and Cytotoxic Activities of Novel Phenacylimidazolium Bromides. Bioorg. Med. Chem. Lett..

[33] Gopalan B., Ke Z., Zhang C., Kng Y., Suhaimi N.-A.M., Riduan S.N., Zhang Y., Zhuo L. (2011). Metal-Free Imidazolium Salts Inhibit the Growth of Hepatocellular Carcinoma in a Mouse Model. Lab. Investig..

[34] Liu L.-X., Wang X.-Q., Zhou B., Yang L.-J., Li Y., Zhang H.-B., Yang X.-D. (2015). Synthesis and Antitumor Activity of Novel N-Substituted Carbazole Imidazolium Salt Derivatives. Sci. Rep..

[35] Xu X.-L., Wang J., Yu C.-L., Chen W., Li Y.-C., Li Y., Zhang H.-B., Yang X.-D. (2014). Synthesis and Cytotoxic Activity of Novel 1-((Indol-3-Yyl)Methyl)–1H-Imidazolium Salts. Bioorg. Med. Chem. Lett..

[36] Shelton K.L., DeBord M.A., Wagers P.O., Southerland M.R., Taraboletti A., Robishaw N.K., Jackson D.P., Tosanovic R., Kofron W.G., Tessier C.A. (2016). Synthesis, Anti-Proliferative Activity, and Toxicity of C4(C5) Substituted N,N′-Bis(Arylmethyl)Imidazolium Salts. Tetrahedron.

[37] DeBord M.A., Southerland M.R., Wagers P.O., Tiemann K.M., Robishaw N.K., Whiddon K.T., Konopka M.C., Tessier C.A., Shriver L.P., Paruchuri S. (2017). Synthesis, Characterization, in Vitro SAR and in Vivo Evaluation of N,N′-Bisnaphthylmethyl 2-Alkyl Substituted Imidazolium Salts against NSCLC. Bioorg. Med. Chem. Lett..

[38] Zhang C.-B., Liu Y., Liu Z.-F., Duan S.-Z., Li M.-Y., Chen W., Li Y., Zhang H.-B., Yang X.-D. (2017). Synthesis and Cytotoxic Activity of Novel Tetrahydrobenzodifuran–Imidazolium Salt Derivatives. Bioorg. Med. Chem. Lett..

[39] Deng G., Zhou B., Wang J., Chen Z., Gong L., Gong Y., Wu D., Li Y., Zhang H., Yang X. (2019). Synthesis and Antitumor Activity of Novel Steroidal Imidazolium Salt Derivatives. Eur. J. Med. Chem..

[40] Stromyer M.L., Southerland M.R., Satyal U., Sikder R.K., Weader D.J., Baughman J.A., Youngs W.J., Abbosh P.H. (2020). Synthesis, Characterization, and Biological Activity of a Triphenylphosphonium-Containing Imidazolium Salt against Select Bladder Cancer Cell Lines. Eur. J. Med. Chem..

[41] Southerland M.R., DeBord M.A., Johnson N.A., Crabtree S.R., Alexander N.E., Stromyer M.L., Wagers P.O., Panzner M.J., Wesdemiotis C., Shriver L.P. (2021). Synthesis, Characterization, In Vitro SAR Study, and Preliminary In Vivo Toxicity Evaluation of Naphthylmethyl Substituted Bis-Imidazolium Salts. Bioorg. Med. Chem..

[42] Chen J.-C., Hsieh Y.-Y., Lo H.-L., Li A., Chou C.-J., Yang P.-M. (2019). In Vitro and In Silico Mechanistic Insights into MiR-21-5p-Mediated Topoisomerase Drug Resistance in Human Colorectal Cancer Cells. Biomolecules.

[43] Yu L.X., Amidon G.L., Polli J.E., Zhao H., Mehta M.U., Conner D.P., Shah V.P., Lesko L.J., Chen M., Lee V.H.L. (2002). Biopharmaceutics Classification System: The Scientific Basis for Biowaiver Extensions. Pharm. Res..

[44] Artursson P., Ungell A., Löfroth J. (1993). Selective Paracellular Permeability in Two Models of Intestinal Absorption: Cultured Monolayers of Human Intestinal Epithelial Cells and Rat Intestinal Segments. Pharm. Res..

[45] Takahashi Y., Kondo H., Yasuda T., Watanabe T., Kobayashi S.-I., Yokohama S. (2002). Common Solubilizers to Estimate the Caco-2 Transport of Poorly Water-Soluble Drugs. Int. J. Pharm..

[46] Cui B., Zheng B.L., He K., Zheng Q.Y. (2003). Imidazole Alkaloids from *Lepidium meyenii*. J. Nat. Prod..

[47] Egorova K.S., Gordeev E.G., Ananikov V.P. (2017). Biological Activity of Ionic Liquids and Their Application in Pharmaceutics and Medicine. Chem. Rev..

[48] Ferraz R., Branco L.C., Prudêncio C., Noronha J.P., Petrovski Ž. (2011). Ionic Liquids as Active Pharmaceutical Ingredients. ChemMedChem.

[49] Balk A., Holzgrabe U., Meinel L. (2015). ‘Pro et Contra’ Ionic Liquid Drugs—Challenges and Opportunities for Pharmaceutical Translation. Eur. J. Pharm. Biopharm..

[50] Pedro S.N., Freire C.S.R., Silvestre A.J.D., Freire M.G. (2020). The Role of Ionic Liquids in the Pharmaceutical Field: An Overview of Relevant Applications. Int. J. Mol. Sci..

[51] Malhotra S.V., Kumar V. (2010). A Profile of the In Vitro Anti-Tumor Activity of Imidazolium-Based Ionic Liquids. Bioorg. Med. Chem. Lett..

[52] Liu F., Ai F., Tian L., Liu S., Zhao L., Wang X. (2016). Infliximab Enhances the Therapeutic Effects of 5-Fluorouracil Resulting in Tumor Regression in Colon Cancer. Oncotargets Ther..

[53] Zhang N., Yin Y., Xu S.-J., Chen W.-S. (2008). 5-Fluorouracil: Mechanisms of Resistance and Reversal Strategies. Molecules.

[54] Balkwill F. (2009). Tumour Necrosis Factor and Cancer. Nat. Rev. Cancer.

[55] Sharma R., Sharma C.L., Mahajan A. (2008). Biological Agents Targeting beyond TNF-Alpha. Indian J. Crit. Care Med..

[56] Bertazza L., Mocellin S. (2010). The Dual Role of Tumor Necrosis Factor (TNF) in Cancer Biology. Curr. Med. Chem..

[57] Grimm M., Lazariotou M., Kircher S., Höfelmayr A., Germer C.T., von Rahden B.H.A., Waaga-Gasser A.M., Gasser M. (2010). Tumor Necrosis Factor-α Is Associated with Positive Lymph Node Status in Patients with Recurrence of Colorectal Cancer—Indications for Anti-TNF-α Agents in Cancer Treatment. Anal. Cell. Pathol..

[58] Szlosarek P., Charles K.A., Balkwill F.R. (2006). Tumour Necrosis Factor-α as a Tumour Promoter. Eur. J. Cancer.

[59] Melling N., Kowitz C.M., Simon R., Bokemeyer C., Terracciano L., Sauter G., Izbicki J.R., Marx A.H. (2016). High Ki67 Expression Is an Independent Good Prognostic Marker in Colorectal Cancer. J. Clin. Pathol..

[60] Edin S., Wikberg M.L., Dahlin A.M., Rutegård J., Öberg Å., Oldenborg P.-A., Palmqvist R. (2012). The Distribution of Macrophages with a M1 or M2 Phenotype in Relation to Prognosis and the Molecular Characteristics of Colorectal Cancer. PLoS ONE.

[61] Forones N.M., Oshima C., Nanogaki S., Tanaka M., Barbosa V. (1999). Determination of proliferative activity using Ki67 and expression of p53 in colorectal cancer. Arq. Gastroenterol..

[62] Ates G., Vanhaecke T., Rogiers V., Rodrigues R.M., Gilbert D.F., Friedrich O. (2017). Assaying Cellular Viability Using the Neutral Red Uptake Assay. Cell Viability Assays.

[63] Carmichael J., DeGraff W.G., Gazdar A.F., Minna J.D., Mitchell J.B. (1987). Evaluation of a Tetrazolium-Based Semiautomated Colorimetric Assay: Assessment of Chemosensitivity Testing. Cancer Res..

