# Supplementary material for: Establishment of In Vitro and In Vivo Anticolorectal Cancer Efficacy of Lithocholic Acid-Based Imidazolium Salts
Source: Int J Mol Sci. 2022 Jun 24;23(13):7019. doi: 10.3390/ijms23137019 (PMC9266680; doi:10.3390/ijms23137019)
Supplement: Supplementary file 1 [file ijms-23-07019-s001.zip › ijms-1767750-supplementary.pdf]

Supplementary data:

Day	Tumor growth (%) - mean ± SD					
	Group					
	**DLD-1**	**DLD-1+ S6**			**DLD-1+ 5-FU**	**DLD-1 + 5-FU + S6 300 mg/kg**
		**100 mg/kg**	**300 mg/kg**	**500 mg/kg**		
1	100 ± 27	100 ± 21	100 ± 21	100 ± 20	100± 23	100 ± 28
5	107 ± 21	116 ± 6	115 ± 2	126 ± 37	112 ± 12	102 ± 24
10	159 ± 57	153 ± 46	181 ± 45	133 ± 65	106 ± 12	94 ± 29
15	190 ± 31	210 ± 74	184 ± 80	159 ± 42 [a]	109 ± 28 [a]	91 ± 30 [a,b]
20	317 ± 87	243 ± 107	196 ± 76	173 ± 55 [a]	104 ± 44 [a]	80 ± 20 [a,b,c,d]
24	541 ± 100	277.71 ± 103.93 [a]	203.03 ± 109.34 [a]	188.53 ± 67.92 [a]	98.25 ± 25.17 [a,b]	76.62 ± 17.95 [a,b,c,d]
28	559 ± 159	282± 112 [a]	206 ± 104 [a]	196 ± 74 [a]	93 ± 19 [a,b]	61 ± 22 [a,b,c,d]

Table S1. Analysis of tumor growth. The results presented the percentage increase in tumor volume, which are expressed as the mean ± SEM for each group. [a] p<0.05 vs the control, [b] p<0.05 vs. DLD-1+100 mg/kg, [c] p<0 vs. DLD-1+300 mg/kg, [d] p<0,05 vs. DLD-1+500 mg/kg.
